# A case of thyrotoxicosis-induced anemia in a patient with painless thyroiditis

**DOI:** 10.1186/s13044-021-00100-6

**Published:** 2021-04-23

**Authors:** Ichiro Komiya, Takeaki Tomoyose, Noriharu Yagi, Gen Ouchi, Tamio Wakugami

**Affiliations:** 1 Department of Internal Medicine, Okinawa Medical Hospital, 2310 Tsuhako-Nishihara, Sashiki, Nanjo, Okinawa 9011414 Japan; 2 Yagi Internal Medicine Clinic, 4-13-9 Kohagura, Naha, Okinawa 9000029 Japan; 3 Department of Hematology, Okinawa Red Cross Hospital, 1-3-1 Yogi, Naha, Okinawa 9028588 Japan; 4 Department of Emergency and Critical Care Medicine, University of Ryukyus Hospital, 207 Uehara, Nishihara, Okinawa 9030215 Japan

**Keywords:** Painless thyroiditis, Anemia, siIL-2R, And thyrotoxicosis

## Abstract

**Background:**

There have been several reports of secondary anemia associated with Graves’ disease. There are no reports of secondary anemia resulting from thyrotoxicosis due to painless thyroiditis (silent thyroiditis). We report the case of a patient with pancreatic diabetes who developed anemia caused by thyrotoxicosis due to painless thyroiditis.

**Case presentation:**

The patient was a 37-year-old man who visited the hospital complaining of fatigue, palpitations, and dyspnea. His hemoglobin was 110 g/l (reference range, 135–176), and mean corpuscular volume was 81.5 fl (81.7–101.6). His free thyroxine (FT4) was high, at 100.4 pmol/l (11.6–21.9); the free triiodothyronine (FT3) was high, at 27.49 pmol/l (3.53–6.14); TSH was low, at < 0.01 mIU/l (0.50–5.00); and TSH receptor antibody was negative. Soluble IL-2 receptor (sIL-2R) was high, at 1340 U/ml (122–496); C-reactive protein (CRP) was high, at 6900 μg/l (< 3000); and reticulocytes was high, at 108 10^9^ /l (30–100). Serum iron (Fe) was 9.5 (9.1–35.5), ferritin was 389 μg/l (13–401), haptoglobin was 0.66 g/l (0.19–1.70. Propranolol was prescribed and followed up. Anemia completely disappeared by 12 weeks after disease onset. Thyroid hormones and sIL-2R had normalized by 16 weeks after onset. He developed mild hypothyroidism and was treated with L-thyroxine at 24 weeks.

**Conclusions:**

This is the first case report of transient secondary anemia associated with thyrotoxicosis due to painless thyroiditis. The change in sIL-2R was also observed during the clinical course of thyrotoxicosis and anemia, suggesting the immune processes in thyroid gland and bone marrow.

## Background

Secondary anemia associated with hyperthyroidism is a relatively rare complication [1]. Many reports have shown that although anemia progresses with hyperthyroidism in Graves’ disease, it is often transient, improving with treatment of Graves’ disease [2–4]. There are no reports of secondary anemia resulting from thyrotoxicosis due to subacute thyroiditis, painless thyroiditis (silent thyroiditis) [5], or other causes. Here, we report a case of transient anemia that developed after the onset of painless thyroiditis in a patient with pancreatic diabetes treated with insulin.

## Case presentation

A 37-year-old man visited our hospital due to gradually progressive fatigue, dyspnea, and palpitations of approximately 14 days duration. His pulse was 104 beats/min, and his body temperature was 36.8 °C. No increase in sweating was observed, goiter was not palpable, and no exophthalmos or ocular movement dysfunction was observed. Laboratory examination showed anemia (Hgb 110 g/l [reference rage, 135–176], mean corpuscular volume [MCV] 81.5 fl [81.7–101.6]), and serum iron (9.5 μmol/l) and ferritin (389 μg/l) were within the normal ranges. Reticulocytes were increased at the time of onset (108 10^9^ /l; reference rage, 30–100), C-reactive protein (CRP) was 6900 μg/l (< 3000), and his haptoglobin was within normal range (0.66 g/l; reference range, 0.19–1.70). The FT4 (free thyroxine) level was high, at 100.4 pmol/l (11.6–21.9), the FT3 (free triiodothyronine) level was high, at 27.49 pmol/l (3.53–6.14), and his TSH was < 0.01 mIU/l (0.50–5.00). TSH receptor antibody, anti-TPO antibody or anti-thyroglobulin antibody were negative. In other clinical tests, soluble interleukin-2 receptor (sIL-2R), measured for differentiation of hematologic malignancies, was high, at 1340 U/ml (122–496); his low-density lipoprotein cholesterol (LDL-C) was low, at 0.78 mmol/l (1.68–3.59); and his high-density lipoprotein cholesterol (HDL-C) was low, at 0.75 mmol/l (1.03–2.48) (Table 1).

**Table 1:** Profile of laboratory data for a diabetes patient with thyrotoxicosis due to painless thyroiditis

Variables	Reference range	Day − 56	Day −28	Day 0	Day 14	Day 28	Day 56	Day 84	Day 112	Day 140	Day 168
Red blood cells, 10^12^/l	4.27–5.70	4.80	4.96	**3.78**	**3.77**	**3.84**	4.50	4.77	4.93	4.88	4.66
Hemoglobin (Hgb), g/l	135–176	144	146	**110**	**107**	**109**	**126**	136	142	146	140
Hematocrit (Hct), /l	0.398–0.518	0.399	0.408	**0.308**	**0.305**	**0.306**	**0.352**	**0.377**	0.392	0.386	0.408
Mean corpuscular volume (MCV), fl	81.7–101.6	83.1	82.3	81.5	80.9	79.7	78.2	79.0	79.5	82.8	83.4
White blood cells (WBC), 10^9^ /l	3.5–9.8	6.4	7.4	5.3	7.2	6.5	8.8	8.6	8.8	5.8	7.4
Platelets, 10^9^ /l	130–369	272	315	254	198	228	289	266	283	322	333
Reticulocytes, 10^9^ /l	30–100			**108**	68						
Thyroid stimulating hormone (TSH), mIU/l	0.50–5.00			**< 0.01**	**< 0.01**	**< 0.01**	**< 0.01**		**10.1**	**15.2**	**18.5**
Free triiodothyronine (FT3), pmol/l	3.53–6.14				**27.49**	**12.61**	**9.37**		3.93	4.35	4.22
Free thyroxine (FT4), pmol/l	11.6–21.9			**100.4**	**91.4**	**51.5**	**33.1**		14.0	13.1	14.0
TSH receptor antibody (TRAb), mIU/l	< 2.0				0.4						
Anti-TPO antibody, IU/ml	< 16				9						
Ant-thyroglobulin antibody, IU/ml	< 28				11						
C-reactive protein (CRP), μg/l	< 3000			**6900**		**12,100**	**5700**	**3700**		1200	
Iron (Fe), μmol/l	9.1–35.5			9.5		9.5					
TIBC, μmol/l	43.2–69.0			34.2		40.8					
Ferritin, μg/l	13–401			389		376	396				
Haptoglobin, g/dl	0.19–1.70			0.66							
Soluble IL-2 receptor (sIL-2R), U/ml	122–496			**1340**		**1010**	**808**		472		365
HDL-C, mmol/l	1.03–2.48	1.27	1.09	**0.75**		**0.98**	1.27	1.42	1.14	1.16	1.14
LDL-C, mmol/l	1.68–3.59	1.99	**1.63**	**0.78**		**1.45**	2.12	2.82	2.87	2.90	2.82
Triglycerides (TG), mmol/l	0.34–1.68	0.46	0.59	0.54		0.84	0.68	0.75	0.84	0.59	0.60
HbA1c (NGSP), %	4.6–6.2	6.9	7.1	7.6		6.5	6.6	6.9	7.7	7.6	7.5
AST, IU/l	13–33	19	18	21		25	30	25	22	27	23
ALT, IU/l	6–30	13	14	19		28	29	21	15	22	21
γGTP, IU/l	10–47	13	14	21		42	**57**	37	26	20	19
Albumin, g/l	40–50	41	42	**31**		**32**	**36**	**36**	**35**	41	41
eGFR, ml/min/1.73 m^2^	> 60	103	97	111		115	104	90	88	79.0	80.0
Drug treatment					Propranolol 30 mg	Propranolol 30 mg					LT4 12.5 μg

Bold data is abnormal value. *eGFR* estimated glomerular filtration rate

**Fig. 1:**
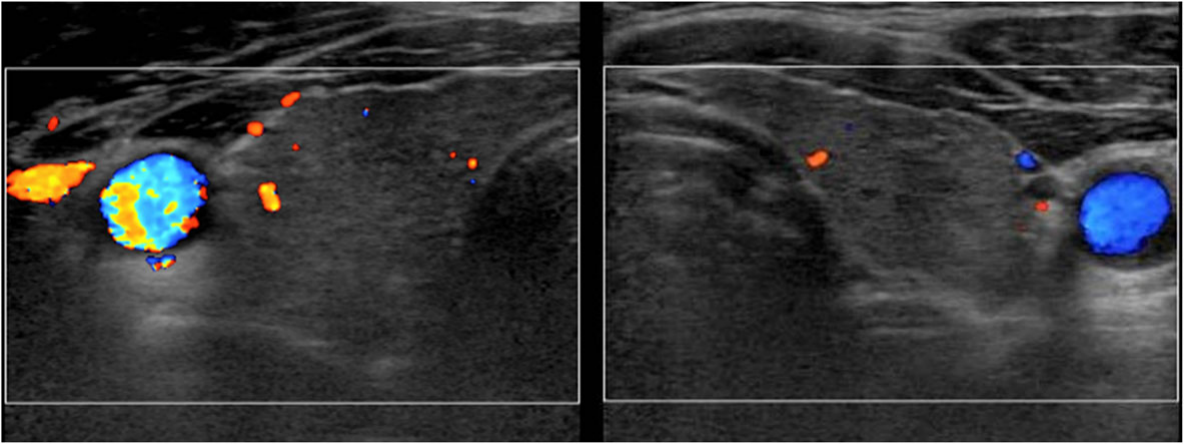
Thyroid ultrasonography findings. Hypoechoic regions are scattered throughout the thyroid gland. No increase in blood flow in the thyroid gland was observed

The patient was previously diagnosed with pancreatic diabetes due to alcoholic pancreatitis at age 25 years, and thus, he was treated with a combination of insulin glargine and insulin aspart. Regular clinical examinations were performed every 28 days, and no abnormalities were found in his biochemical data or complete blood count, other than his plasma glucose and HbA1c. Propranolol (30 mg/day) was prescribed at day 14 and stopped on day 56. Thyroid ultrasonography was performed on day 28, and hypoechoic regions were observed throughout the thyroid gland (Fig. 1).

**Fig. 2:**
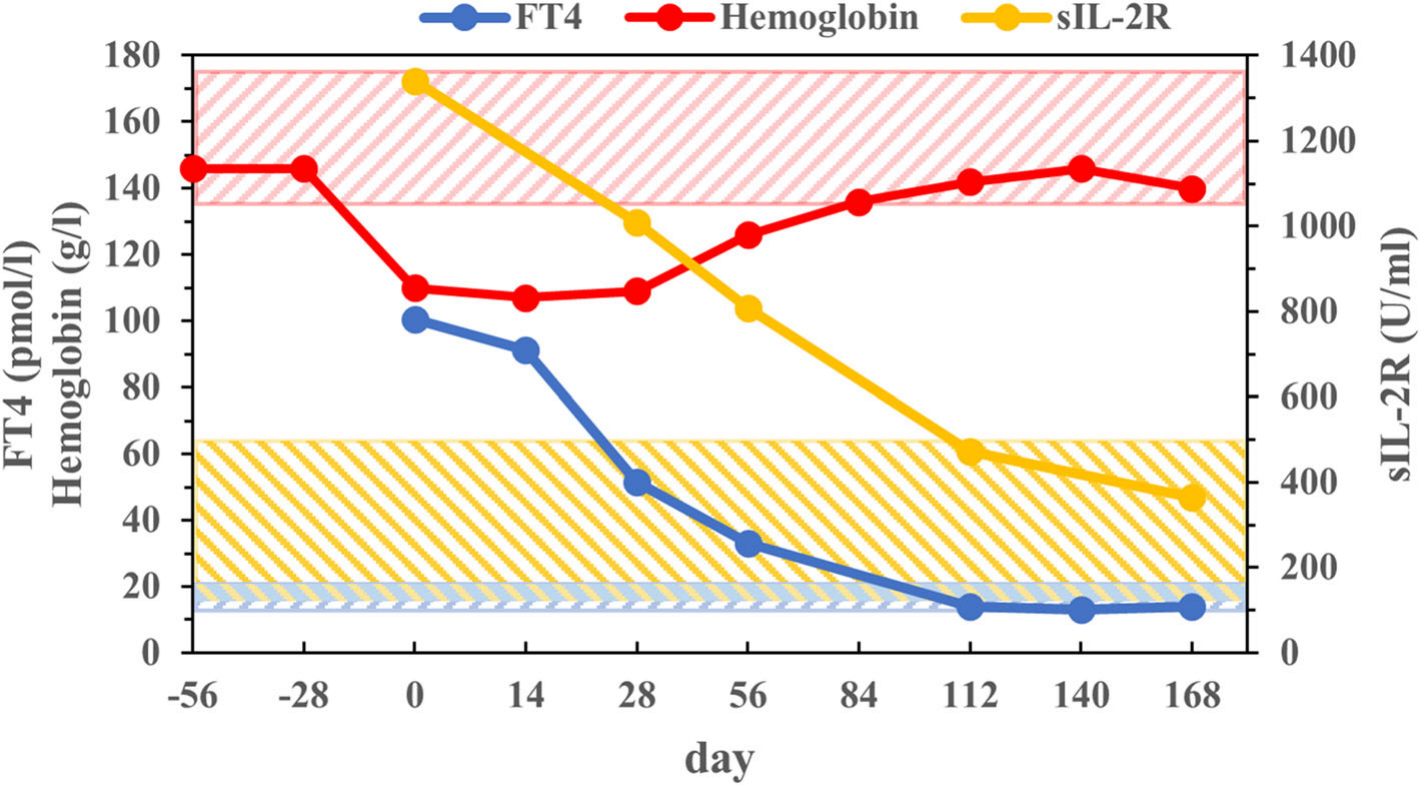
Profile of FT4, hemoglobin, and sIL-2R. The patient’s hemoglobin had normalized by day 84. FT4 and sIL-2R returned to be normal by day 112. The shaded area shows the reference range for each variable

The anemia disappeared by day 84. The patient’s thyroid hormones and sIL-2R normalized by day 112, and CRP normalized by day 140. The clinical courses of Hgb, FT4, and sIL-2R were shown in Fig. 2. There was slight decrease in LDL-C and HDL-C, and increases in liver enzymes at disease onset, but these changes disappeared by day 140 (Table 1). His serum albumin was low at onset but normalized by day 140. The patient’s insulin regimen was not changed over the entire clinical course. He developed mild hypothyroidism on day 112 and was started on 12.5 μg of L-thyroxine replacement therapy on day 168 (Table 1).

## Discussion and conclusions

We reported a case of painless thyroiditis-induced thyrotoxicosis that suddenly led to anemia within 4 weeks after the patient’s last visit to our hospital. Although it was not possible to prove reduced iodine uptake using thyroid scintigram, negative TSH receptor antibody, the mild increase of CRP, lack of tenderness in thyroid gland, and the diffuse destructive findings on thyroid ultrasonography indicated painless thyroiditis [5]. In addition, FT3/FT4 ratio (0.301) was low, suggesting painless thyroiditis, because the median (IQR) of FT3/FT4 ratio in painless thyroiditis was reported as 0.310 (0.203–0.608) [6]. This is the first report of secondary anemia associated with painless thyroiditis. Although the levels remained within the normal range, slight decrease in WBC and platelets presented at disease onset [1]. Anemia, and the decrease in WBC and platelets completely disappeared by 12 weeks after onset, with spontaneous remission of thyrotoxicosis.

In addition to the clinical course of thyrotoxicosis and associated anemia, the present case provided interesting laboratory data. First, sIL-2R was high at onset but normalized by 16 weeks after onset of anemia. Elevated levels of sIL-2R have been reported in hyperthyroidism of Graves’ disease and it was suggested that thyroid hormones directly enhance sIL-2R production in lymphocytes [7]. The levels in sIL-2R also increased in patients with the thyrotoxicosis due to painless thyroiditis [8]. Second, LDL-C and HDL-C levels decreased due to thyrotoxicosis. Excessive thyroid hormone levels lower serum LDL-C and HDL-C levels via several mechanisms [9–11]. We previously reported that increased sIL-2R cause significant decreases levels in HDL-C and LDL-C in patients with hematologic malignancies [12]. Increased cytokines were recently reported to be associated with hypolipidemia in COVID-19 patients [13]. Presumably, increases in levels of both thyroid hormones and sIL-2R are associated with the decreases in HDL-C and LDL-C.

Painless thyroiditis (silent thyroiditis) is a self-limiting inflammatory disorder of the thyroid gland characterized by an early thyrotoxicosis phase caused by the release of thyroid hormones and a late hypothyroidism phase, with complete resolution in most cases [5, 14]. The pathophysiologic mechanism of painless thyroiditis is unknown, but the possibility of immune disorder involvement has been suggested [5, 7, 8, 15]. Painless thyroiditis generally manifests as a lymphocyte infiltration of the thyroid follicles, causing thyroid follicular cell damage [5]. In our case, we observed a typical course of painless thyroiditis. The secondary anemia caused by thyrotoxicosis has improved, but we would like to carefully follow up on the continuation of thyroid hormone replacement therapy.

The mechanism by which anemia develops in thyrotoxicosis is not clear. Shortened erythrocyte survival or ineffective erythropoiesis have been suggested as potential causes of anemia in thyrotoxicosis [2–4]. Moreover, slight decrease in WBC and platelets was observed in present case, which could have been due to a variety of mechanisms. The involvement of autoantibodies in leukocytes and platelets resulting in increased destruction of hematopoietic cells by immunological mechanisms has been reported [16]. The increase in sIL-2R, which had been increasing at the onset of painless thyroiditis, may suggest the result of immune process abnormality rather than the increase in thyroid hormones [8, 17]. The involvement of immune processes in the onset of painless thyroiditis could help explain the pathophysiology of anemia [18]. In present case, however, both anti-TG and anti-TPO antibodies were negative. Anti-thyroglobulin and anti-TPO antibodies prevalence in painless thyroiditis have been reported to be 70 and 30%, respectively [19]. The patient had an history of acute pancreatitis. It is interesting to assume the immune mechanisms as the pathogenesis of acute and chronic pancreatitis [20], and it is natural to assume that the immune mechanism was involved in the development of silent thyroiditis and anemia in present case.

In conclusion, we reported the case of a diabetes patient with secondary anemia resulting from thyrotoxicosis. Thyrotoxicosis was caused by painless thyroiditis, but there have been no reports of secondary anemia induced by painless thyroiditis. The change in sIL-2R was also observed during the clinical course of thyrotoxicosis and anemia.

## Data Availability

The collection of data that supports the findings in this study is available from Okinawa Medical Hospital. Data are available from the authors upon reasonable request and with permission of Okinawa Medical Hospital.

## Abbreviations

TSH: Thyroid stimulating hormone
FT4: Free thyroxine
FT3: Free triiodothyronine
sIL-2R: Soluble interleukin-2 receptor
HDL-C: High-density lipoprotein cholesterol
LDL-C: Low-density lipoprotein cholesterol
